# The Relationship between Impulsive Choice and Impulsive Action: A Cross-Species Translational Study

**DOI:** 10.1371/journal.pone.0036781

**Published:** 2012-05-04

**Authors:** Nienke Broos, Lianne Schmaal, Joost Wiskerke, Lennard Kostelijk, Thomas Lam, Nicky Stoop, Lonneke Weierink, Jannemieke Ham, Eco J. C. de Geus, Anton N. M. Schoffelmeer, Wim van den Brink, Dick J. Veltman, Taco J. de Vries, Tommy Pattij, Anna E. Goudriaan

**Affiliations:** 1 Department of Anatomy and Neurosciences, Neuroscience Campus Amsterdam, VU University Medical Center, Amsterdam, The Netherlands; 2 Department of Psychiatry, Academic Medical Center, University of Amsterdam, Amsterdam, The Netherlands; 3 Department of Biological Psychology, VU University, Amsterdam, The Netherlands; 4 Department of Psychiatry, VU University Medical Center, Amsterdam, The Netherlands; Université Pierre et Marie Curie, France

## Abstract

Maladaptive impulsivity is a core symptom in various psychiatric disorders. However, there is only limited evidence available on whether different measures of impulsivity represent largely unrelated aspects or a unitary construct. In a cross-species translational study, thirty rats were trained in impulsive choice (delayed reward task) and impulsive action (five-choice serial reaction time task) paradigms. The correlation between those measures was assessed during baseline performance and after pharmacological manipulations with the psychostimulant amphetamine and the norepinephrine reuptake inhibitor atomoxetine. In parallel, to validate the animal data, 101 human subjects performed analogous measures of impulsive choice (delay discounting task, DDT) and impulsive action (immediate and delayed memory task, IMT/DMT). Moreover, all subjects completed the Stop Signal Task (SST, as an additional measure of impulsive action) and filled out the Barratt impulsiveness scale (BIS-11). Correlations between DDT and IMT/DMT were determined and a principal component analysis was performed on all human measures of impulsivity. In both rats and humans measures of impulsive choice and impulsive action did not correlate. In rats the within-subject pharmacological effects of amphetamine and atomoxetine did not correlate between tasks, suggesting distinct underlying neural correlates. Furthermore, in humans, principal component analysis identified three independent factors: (1) self-reported impulsivity (BIS-11); (2) impulsive action (IMT/DMT and SST); (3) impulsive choice (DDT). This is the first study directly comparing aspects of impulsivity using a cross-species translational approach. The present data reveal the non-unitary nature of impulsivity on a behavioral and pharmacological level. Collectively, this warrants a stronger focus on the relative contribution of distinct forms of impulsivity in psychopathology.

## Introduction

Impulsivity is a hallmark and common feature in various psychiatric disorders, including substance use disorder, attention deficit hyperactivity disorder (ADHD), conduct disorder, bipolar disorder, pathological gambling and personality disorders [1]. Although impulsivity can be broadly defined as behavioral actions without adequate forethought, there is growing evidence that impulsivity is no unitary construct, but rather is dissociable into different aspects reflecting distinct underlying cognitive, emotional, and neural processes [2]. Nonetheless, detailed research on the relationship between various aspects of impulsivity is still scarce.

Two widely recognized behavioral phenomena of impulsivity are impulsive choice and impulsive action. *Impulsive choice* is oftentimes operationalized by impulsive decisions resulting from a distorted evaluation of delayed consequences of behavior and an increased preference for (smaller) immediate rewards over more beneficial delayed rewards. On the other hand, *impulsive action* reflects the failure to inhibit an inappropriate response to prepotent stimuli [2]–[4].

In addition to self-report measures, impulsive choice and impulsive action can be assessed in different behavioral paradigms. Importantly, for most of these behavioral paradigms similar versions exist for humans and laboratory animals. In humans delay discounting paradigms are generally used to assess impulsive choice [5]. To measure impulsive action, the go-no go task, stop signal task, Stroop task, or commission errors during a continuous performance task (CPT) are most often utilized in humans [6]. Preclinical laboratory animal researchers have developed translational analogies of these neuropsychological tasks such as the delayed reward task (DRT) to study impulsive choice and the go-no go task, stop signal reaction time task and the five-choice serial reaction time task (5-CSRTT) to measure impulsive action (for review see [7]). Translational, cross-species approaches combining clinical and preclinical data on impulsivity are particularly suited to deepen our understanding of the neurobiological mechanisms underlying impulsivity and the multidimensional nature thereof and may ultimately lead to improved treatment strategies for psychiatric disorders characterized by maladaptive impulsivity.

In recent years, both animal (for reviews see [4], [8], [9]) and human (for reviews see [10], [11]) research has tremendously contributed to an increased understanding of the neurobiological mechanisms of impulsivity and has indicated that on a neurobiological level there is partial overlap in the neurotransmitter systems and brain regions modulating impulsive choice and impulsive action. In addition, the involvement of these forms of impulsivity in psychopathology, for example ADHD [12] and drug dependence [13]–[17], show both overlap as well as dissociation.

Despite accumulating evidence further supporting the view that impulsivity is not a unitary construct, to date there is, especially in the preclinical animal literature, only limited data available on within-subject comparisons of various aspects of impulsivity. This approach is particularly suited to examine the multidimensional nature of impulsivity, because, in contrast to a between-subjects comparison, potential findings of separable aspects of impulsivity cannot be attributed to individual differences that might exist between subjects. Nonetheless, to date, most rodent work is conducted in separate groups each performing a single impulsivity paradigm and findings from the few rodent studies that have tested both impulsive action and choice in the same animals have been inconsistent: It has been demonstrated that animals showing high levels of impulsive action, also display steep discounting behavior [18], whereas such a relationship is not detected in other studies [19], [20]. In healthy volunteers, the studies that employed a within-subjects design have generally revealed separate factors for impulsive choice and impulsive action [3], [21]–[24]. The inconsistent findings in rodents and the limited number of studies using within-subject approaches warrant further investigation of the multidimensional nature of impulsivity in rodents and the translational value to human data.

The current study aimed to investigate the interrelationship between impulsive choice and impulsive action in a cross-species translational (rats and humans) design, using multiple assessments within the same subjects. To this aim, a cohort of rats was trained in the DRT and 5-CSRTT paradigm, the most often used behavioral laboratory measures for impulsive choice and impulsive action. In parallel, a cohort of healthy volunteers performed analogous impulsivity measures, namely a delay discounting task (DDT) for impulsive choice and immediate and delayed memory task (IMT/DMT, a modified CPT) for impulsive action. Additionally, to further delineate the interrelationship between aspects of impulsivity, human subjects completed the stop signal task (SST); one of the most frequently used paradigms for impulsive action in human studies, and the self-report Barratt Impulsiveness Scale (BIS-11). To extend previous neurobiological findings on the various aspects of impulsivity based on between-subject approaches, pharmacological challenges with the clinically relevant psychostimulant amphetamine (AMP) and the norepinephrine reuptake inhibitor atomoxetine (ATO) were conducted in the rodent experiment. Using this within-subjects, translational approach, we aimed to establish whether impulsive choice and impulsive action represent separate dissociable aspects or a unified construct of impulsivity in rats and humans.

## Methods

### Rodent study

#### Subjects

Thirty male Wistar rats (Harlan, Horst, The Netherlands), initially weighting 240–270 grams, were pair-housed in Macrolon cages on a reversed 12 hour day/night cycle (lights on 7 PM) in a temperature (21±2°C) and humidity (50±10%) controlled room. Behavioral testing was conducted during the dark phase of the day/night cycle. Rats were food restricted (maintained at about 85%–90% of their free feeding weight) by feeding them 12 (weekdays) or 14 (weekend) gram of regular chow per day per rat. Water was available ad libitum. Experiments were approved by the Animal Care committee of the VU University, Amsterdam, The Netherlands (protocol number ANW09-10).

#### Impulsive choice: Delayed Reward Task (DRT)

Rats were trained in the DRT. Apparatus and procedure were similar to previous studies [25], but with levers instead of nose-poke holes, and are described in detail in the Methods S1. In short, the final procedure of the DRT was as follows: Rats were placed in an operant chamber containing a food receptacle and retractable levers with cue lights above on both sides of the receptacle. Left and right cue lights were illuminated, levers extended and a lever press at one side resulted in an immediate delivery of 1 food pellet, whereas pressing the other lever resulted in a delayed delivery of 4 food pellets. The delay of the larger reward was increased within the session from 0, 5, 10, 20 to 40 s, per block of 12 trials. Behavioral outcome measures were: preference for the large reward, number of omissions during choice trials and the indifference point (Indifference point = Preference at delay 0/(1+k*delay)) [26]. In order to allow a direct comparison with the human study, a log transformed k-value was used as an additional measure of impulsive choice.

#### Impulsive action: 5-choice serial reaction time task (5-CSRTT)

Detailed descriptions of apparatus and procedure were provided previously [27] and are included in the Methods S1. In short, the final procedure of the 5-CSRTT was as follows: Rats were placed in an operant chamber containing a food receptacle and an array of 5 rectangular apertures in the opposing wall. After starting the trial by a nose poke in the receptacle, they were required to wait for 5 s (inter-trial interval, ITI) before one of the lights within the apertures were illuminated for 1 s. A nose-poke response in this illuminated hole was rewarded with one food pellet. Every session consisted of 100 trials or lasted 30 min, whichever occurred first. Attentional performance was defined by accurate choice, the number of omissions and reaction times. Premature responses, made during the ITI, were the measure of impulsive action. Besides the regular sessions containing an ITI of 5 s, the ITI was lengthened to 7 s in 3 sessions with one week of ITI 5 s sessions in between. These challenge sessions are often performed to increase premature responding and to differentiate between high and low impulsive animals (e.g. [28]).

#### Drugs

Both amphetamine sulfate (AMP; OPG, Utrecht, The Netherlands) and atomoxetine hydrochloride (ATO; Tocris Bioscience, Bristol, UK) were dissolved in sterile saline and injected intraperitoneally in a volume of 1 ml/kg body weight. AMP (0.5 mg/kg) and ATO (1 mg/kg) were injected 20 and 45 minutes before testing, respectively. These doses were chosen based on their robust effect in previous studies employing the same behavioral tasks (e.g. [18], [27], [25]).

#### Design

Half of the animals was first trained in the DRT, the other half first in the 5-CSRTT. After training in either of the tasks, baseline behavior was calculated as the average behavior of the last three sessions of one week. To perform the pharmacological challenges, rats were trained on Mondays and Tuesdays in task one, and on Thursdays and Fridays in task two. As soon as animals showed stable baseline behavior in both tasks, drugs were tested on Tuesdays and Fridays using a latin-square design.

#### Statistical analysis

Pearson's correlation analyses were performed between the baseline impulsivity measures and the responsivity to the drug challenges (performance under drug – saline) in the DRT and 5-CSRTT. The impulsivity measure in the DRT was the indifference point and the k-value and in the 5-CSRTT the number of premature responses reflected impulsive behavior. Pharmacological effects on impulsive choice in the DRT were analyzed with repeated measures ANOVA, with drug (AMP or ATO) and delay (0–40) as within subject factors. Omissions during choice trials in the DRT and all parameters of the 5-CSRTT were analyzed using paired samples T-tests for AMP and ATO compared to saline. Data were analyzed using the Statistical Package for the Social Sciences (SPSS, Chicago, IL, USA) version 16.0 and the significance level was set at p<0.05.

### Human study

#### Subjects

Subjects were 101 healthy students (78 females, 23 males: 21.20 yr (SD = 2.39)), recruited through posted advertisements. Exclusion criteria were any medication other than oral contraception and presence of a neurological, medical or psychiatric disorder. All subjects were screened for the presence of Axis I psychiatric disorders using the Mini International Neuropsychiatric Interview plus (MINI-plus) [29]. The study was approved by the Medical Ethical Committee of the University of Amsterdam (approval number: METC 10/264 #10.17.2070) and written informed consent was obtained from all participants.

**Figure 1:**
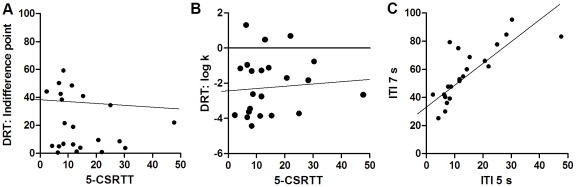
Correlation between impulsive choice and action in rats. In rats (n = 22), there was no correlation between impulsive action, based on premature responses in the 5-CSRTT, and impulsive choice, based on (A) the indifference point (r = −.22) or (B) the log k-value (r = .09) in the DRT. Within the 5-CSRTT (C) there was a correlation (r = .77) between impulsive action with a standard inter trial interval (ITI 5 s) and lengthened inter trial interval (ITI 7 s).

#### MeasImpulsive choice: Delayed Discounting Task (DDT)

A DDT was used to measure impulsive choice [30]. Several trials were presented in which the subject had to choose between a “standard” and an “alternative” item. The standard item was €10.00 to be received after a certain delay (0, 7, 30, 90, 180 or 365 days) and the alternative item was an amount of money (€0.01, €0.25, €0.50, and further amounts increasing in 0.50 increments up to €10.50) to be received immediately. All possible combinations of six standard and 23 alternative items add up to 138 questions in total. However, one question would require subjects to choose between two identical choices. This same-items question was excluded, hence subjects answered 137 questions. Standard and alternative items were presented in a random order and without replacement. Subjects made their choice by clicking the left mouse button on the item they preferred. They were able to change their preference by clicking on the other item, before confirming their choice by clicking on “next question”. The subjects were asked to make their own personal choice as if the rewards were real and informed that there was no right or wrong answer. Standard and alternative items were presented at random order and without replacement. The order (first or second) in which the standard and alternative items were presented within each question was also randomized. Indifference points were derived for each delay, reflecting the point at which the preference switched from the larger later reward to the smaller immediate reward. In most cases, the indifference point was discrete. However, when it was not, the indifference point was defined as the point midway between the lowest value of the alternative at which the subject chose the alternative item for two consecutive, descending values and the highest value of the standard for which the subject chose the standard item for two consecutive ascending values. Indifference points across the delays were analyzed using the hyperbolic decay function yielding k-values representing the rate of discounting (referred to as DDT k value) [26]. Higher k values reflect greater discounting by delay and therefore indicate greater impulsivity.

#### Impulsive action: Immediate and Delayed Memory Task (IMT/DMT)

The human analogue of the preclinical 5-CSRTT is the Continuous Performance Task (CPT). Because the CPT was primarily developed for use with children or severely impaired populations, using a standard CPT might result in ceiling effects in the current sample. Therefore, we included the IMT/DMT developed by Dougherty and colleagues [31], which is a modified (more demanding) CPT that has been validated to measure impulsive action in healthy subjects [32]. The IMT/DMT consists of two task components (IMT and DMT) that each feature two 5 min blocks. The order of the blocks was the same for each subject (ie, IMT/DMT/IMT/DMT) and blocks were separated by a 30 sec rest period, resulting in a total test duration of 21.5 min. Both the IMT and DMT consisted of randomly generated 5-digit numbers (e.g. 27394) displayed on a computer screen in black on a white background. In the IMT, these numbers each appear for 500 ms, followed by a blank screen for 500 ms, followed by the next 5-digit numbers. Subjects are required to respond when two identical numbers are presented in sequence (target stimuli). In the DMT a distracter stimulus (the number 12345, which is to be ignored) appears 3 times between each of the target numbers. Responses to targets were recorded as correct detections. Responses to a non-identical number were recorded as either a commission error, if the number differed from the target on only 1 digit (termed a catch), or a filler error, if the number differed from the target on more than one digit (termed a filler). The appearance probability for filler stimuli was 34%, and 33% for either target or catch stimuli. The primary dependent impulsive action measure for both IMT and DMT is the ratio of commission errors to correct detections (IMT or DMT Ratio).

#### Impulsive action, additional measure: Stop Signal task (SST)

In humans, impulsive action is most often measured using either a stop signal or a go-no go paradigm. We therefore included a SST [33], [34] to investigate whether the ratio of commission errors to correct detections on the IMT/DMT and performance on the SST measure the same construct, namely impulsive action. The stop signal task consisted of 252 randomized trials with a Go/Stop ratio of 80/20 and lasted approximately 12 min. Before each trial a small cross appeared on the screen for 500 ms to engage attention, immediately followed by an airplane (facing to the left or to the right) presented for 1000 ms (Go trial). The ITI varied between 1 and 2 s. During the Go trials the subjects had to respond by pressing a button with their left or right index fingers when the airplane faced to the left or right, respectively. The subjects were instructed to respond as fast and accurate as possible. Occasionally (20% of the times), the Go stimulus was followed by a Stop stimulus (a cross) projected over the Go stimulus. The subjects were instructed to try to withhold the Go response (pressing a button) when seeing the Stop signal. By adjusting the interval between the Go stimulus and the Stop stimulus, the Stop Signal Delay (SSD), the difficulty of stopping was varied. The SSD varied using a staircase procedure: a failed stop trial reduced the SSD, making it easier to inhibit the Go response during the next stop trial. A successful Stop increased the subsequent SSD, making it more difficult to succeed in the next Stop trial. This staircase procedure converged upon a critical SSD, which represents the time delay required for a subject to successfully stop a response on approximately 50% of the Stop trials. The time required for the stop signal to be successfully processed, the Stop Signal Reaction Time (SSRT), was computed by subtracting the critical SSD from the median Go reaction time [33]. The SSRT is the time required for a subject to inhibit his response after seeing the Stop Signal corrected for mean reaction time to Go trials. A short SSRT indicates good response inhibition and a longer SSRT indicates poorer response inhibition.

#### Self report impulsivity questionnaire: Barratt Impulsiveness Scale (BIS-11)

In order to evaluate the overlap between behavioral and self-report measures of impulsivity, a Dutch version of the BIS-11 [35] was included, a 30-item questionnaire measuring impulsivity. Each item was measured on a 4-point scale (rarely/never, occasionally, often, almost always), with higher scores indicative of greater impulsivity. For factor analysis scores on the cognitive, motor and non-planning subscales were used.

#### Design

During a test session, lasting approximately 2 hours, subjects filled out questionnaires and performed all three laboratory measures of impulsivity in a semi-counterbalanced order such that the IMT/DMT and Stop tasks were never presented consecutively because of task demands. After completing the tasks, subjects were debriefed and reimbursed with 20 Euros.

#### Statistical analyses

Because the k-values derived from the delay discounting task were not normally distributed, k-values were first log-transformed. Similar to the animal study, Pearson's correlation analysis was performed between the impulsivity scores of the DDT and IMT/DMT. The level of significance was set at p<0.05 (two-tailed). In addition, principal component analysis using a varimax rotation with Kaiser Normalization was performed using all measures of impulsivity. Components with eigenvalues ≥1 were retained and component loadings of ≥0.5 within identified components were considered relevant. All analyses were performed using SPSS (Chicago, IL, USA) version 16.0.

## Results

### Rodent study

Of all thirty rats, in the DRT, five animals consistently chose the big reward, independent of delay and in the 5-CSRTT, three animals showed on average more than 40 omissions per session. These animals were therefore excluded from further analyses. Baseline impulsivity in the DRT as well as the 5-CSRTT was independent of the order of training: impulsive choice [indifference point: T(20) = −1.32, ns; log K: T(20) = 1.08, ns] and premature responses [T(20) = .33, ns].

At baseline, there was no correlation between impulsive action, defined by the number of premature responses and impulsive choice, defined by the indifference point [r(22) = −.22, ns] or log k-value [r(22) = .09, ns] (see figure 1A and 1B). Within the 5-CSRTT, however, there was a strong correlation between premature responses made under standard and lengthened, 7 s, ITI conditions [r(22) = .77, p<0.001] (see figure 1C).

**Figure 2:**
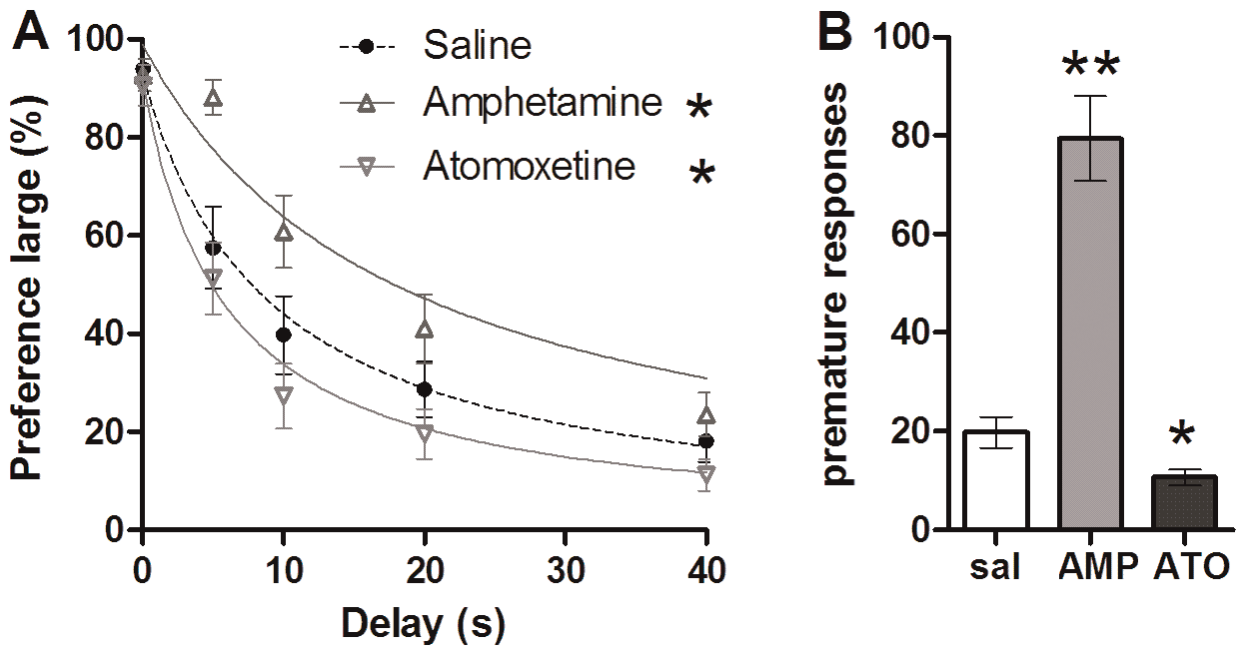
Pharmacological manipulation of impulsive choice and action in rats. In rats (n = 22), the preference for the large reward in the DRT decreased with increasing delays (A) and amphetamine (0.5 mg/kg) decreased impulsive choice in rats, whereas atomoxetine (1 mg/kg) increased impulsive choice. In the 5-CSRTT (B), amphetamine increased premature responding, whereas atomoxetine decreased the number of premature responses. *p<0.05, **p<0.001 compared to vehicle.

**Figure 3:**
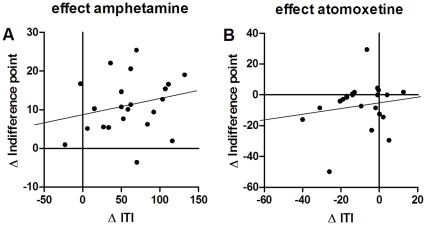
Correlation between impulsive choice and action after pharmacological manipulations in rats. In rats (n = 22), there was no correlation between the effects of (A) amphetamine (0.5 mg/kg, r = .22) and (B) atomoxetine (1 mg/kg, r = .21) on the two impulsivity measures: the Δ indifference point ( = drug challenge minus vehicle) of the delayed reward task and the Δ premature responses ( = drug challenge minus vehicle) in the 5-choice serial reaction time task did not correlate.

In the DRT (see figure 2A), compared to vehicle, AMP caused a decrease in impulsive choice behavior, reflected in an increased preference for the large delayed reward over increasing delays [drug*delay: F(4,84) = 5.84, p<0.001; delays T(21) = 0: .037, 40: −2.00, ns, 5: −3.96, 10: −3.77, 20: −2.38, p<0.05]. In contrast, ATO increased impulsive choice, by reducing the preference for the large reward at all delays [drug: F(1,20) = 6.95, p<0.05]. Neither AMP [T(21) = −.14, ns], nor ATO [T(20) = −.79, ns] had an effect on the number of omissions (see table S1). In the 5-CSRTT, as shown in figure 2B, impulsive action was increased by AMP [T(21) = −6.83, p<0.001] and decreased by ATO [T(20) = 3.27, p<0.05. Neither AMP [T(21) = .39, ns], nor ATO [T(20) = −1.24, ns] changed the number of omissions. Accuracy was decreased by AMP [T(21) = 2.92, p<0.05], whereas ATO [T(20) = −1.38, ns] had no effect (see table S2). Correct response latency was not affected by AMP [T(21) = 1.66, ns] or ATO [T(20) = −1.63, ns]. Finally, correlation analyses revealed that the pharmacological effects (impulsivity under drug – vehicle) of both AMP [ITI-indifference point: r(22) = .22, ns; ITI-logK: r(22) = −.29, ns] and ATO [ITI-indifference point: r(22) = .21, ns; ITI-logK: r(22) = −.12, ns] on the two measures of impulsivity were not related to each other (see figure 3).

### Human study

**Figure 4:**
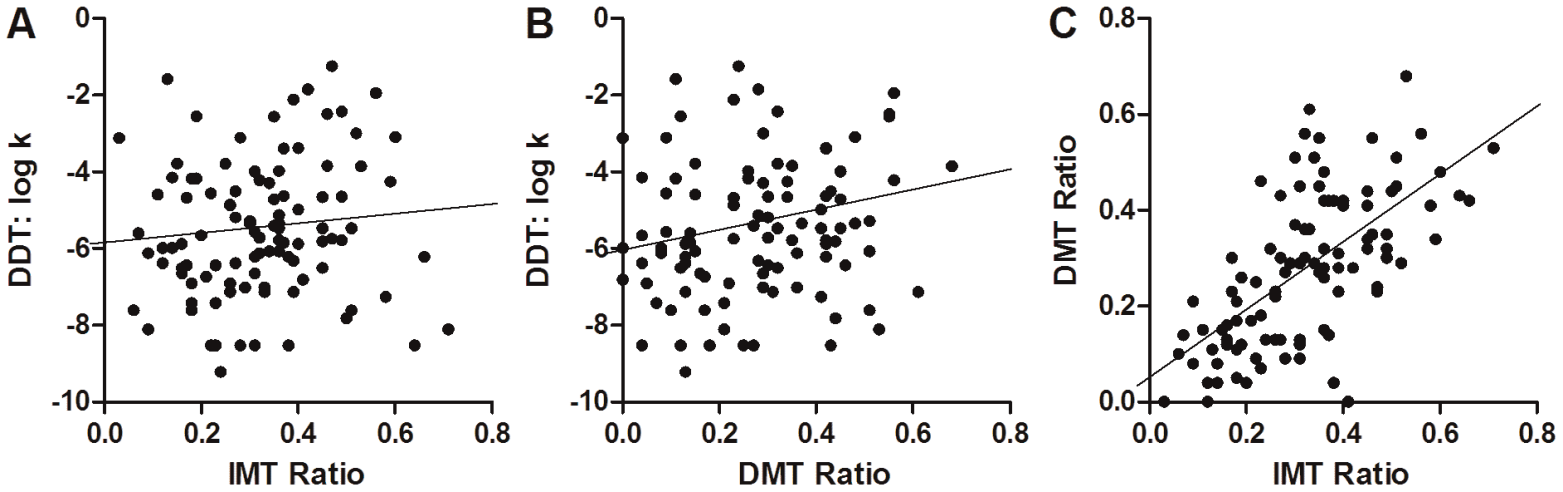
Correlation between impulsive choice and action in humans. In humans (n = 100), there was no correlation between impulsive choice (log DDT k value) and impulsive action measured as the ratio of commission errors to correct detections in (A) IMT (r = .11) and (B) DMT (r = .16). Within the IMT/DMT (C) there was a correlation between the ratio of commission errors to correct detections in the IMT and DMT (r = .64).

In the human study, one subject was excluded because of a performance below chance level on the DMT. The IMT Ratio and DMT Ratio (i.e. the ratio of commission errors to correct detections which are indices of impulsive responding in the IMT and DMT), correlated positively with each other [r(100) = .64, p<0.001], but, there was no correlation between either the IMT Ratio [r(100) = .11, ns] or the DMT Ratio [r(100) = .16, ns] with the DDT k value (obtained by a hyperbolic decay function representing discounting rate, see figure 4). In addition, the subscales of the BIS-11 questionnaire correlated positively with each other (cognitive and motor: r(100) = .42, p<0.001; cognitive and non-planning: r(100) = .34, p<0.001; motor and non-planning: r(100) = .38, p<0.001). The DMT Ratio showed a weak but significant positive correlation with impulsive responding in the Stop Signal Task reflected by the Stop SSRT [r(100) = .20, p = 0.04]. No other correlations between the impulsivity measures were found (see table S3).

The principal component analysis with all impulsivity measures yielded three principal components with eigenvalues ≥1, which together accounted for 65.3% of the variance (see table 1). For the first component, loadings were only significant for the three BIS-11 subscales. The second component had significant positive loadings only for the IMT, DMT and the SSRT. The third principal component had significant positive loadings only from the DDT and a negative loading of the SSRT. The demographics and mean scores on all measures of impulsivity of the sample are described in table S4.

**Table 1:** Principal component analysis yielding 3 rotated components *(N = 100)*.

	Rotated Components		
	1	2	3
Eigenvalues	1.79	1.77	1.07
Variance	25.55%	25.23%	15.33%
DDT k value^a^	−0.04	0.24	**0.74**
IMT Ratio^b^	0.04	**0.85**	0.07
DMT Ratio^b^	−0.01	**0.87**	0.10
Stop SSRT	0.03	0.48	**−0.55**
BIS-11 cognitive impulsivity	**0.78**	−0.08	−0.24
BIS-11 motor impulsivity	**0.79**	0.05	−0.05
BIS-11 nonplanning impulsivity	**0.73**	0.09	0.39

Factor loadings >0.5 as significant.

a k values were obtained by a hyperbolic decay function and log transformed.

b IMT and DMT scores were calculated as the ratio of commission errors to correct detections.

*DDT: Delay Discounting Task, IMT: Immediate Memory Task, DMT: Delayed Memory Task, SSRT: Stop Signal Reaction Time, BIS-11: Barratt Impulsiveness Scale.*

## Discussion

Using a within-subjects, cross-species translational approach, the current study showed that impulsive choice and impulsive action appear not correlated in both rats and humans. Moreover, in rats, behavioral responsivity to pharmacological challenges with amphetamine and atomoxetine did not correlate in both paradigms. In addition, in healthy volunteers, self-reported impulsivity was not associated with behavioral measures. Likewise, impulsive choice differed from the two measures of impulsive action. Together, these findings provide further support for the notion that impulsivity is not a unitary but rather a multifaceted construct in both rats and humans.

### Impulsive choice and action are unrelated in rats

The results of our rodent study revealed no correlation between impulsive action, measured in the 5-CSRTT, and impulsive choice, measured in the DRT. Thus, these data underscore that impulsive choice and impulsive action are separable at a behavioral level. To date, there are only two earlier reports on the multidimensional aspects of impulsivity using these behavioral paradigms in a within-subjects approach. The present results, obtained in Wistar rats, are consistent with previous results showing no correlation between impulsive choice, measured with the DRT, and the one-choice visual attention task in Lister Hooded rats [20]. In contrast, in another study [18] Lister Hooded rats that displayed high impulsive action also showed a steeper delay discounting curve on the DRT compared to low impulsive individuals. Nonetheless, the absence of correlational analyses on the two impulsivity measures hampers a direct comparison with the present data.

The observation that impulsive choice and impulsive action under baseline conditions were separable aspects in the present study was corroborated by a comparison of drug-induced changes in impulsivity. Importantly, the within-subjects behavioral effects of both atomoxetine and amphetamine on impulsivity measures in 5-CSRTT and DRT did not correlate. These dissociable pharmacological effects strongly suggest different underlying neural correlates of impulsive choice and impulsive action, similar to previous between-subjects studies showing opposing effects of amphetamine on impulsive action and impulsive choice [36]–[38]. Thus, these findings seem in line with earlier observations showing dissociable roles of for example dopamine, glutamate and serotonin in modulating impulsive choice and impulsive action (for reviews see [4], [8], [9]). The data obtained with atomoxetine in the 5-CSRTT are in line with previous preclinical findings [39]–[41]. Surprisingly, in contrast to earlier work showing a decrease [41] or null effects [42] of atomoxetine on impulsive choice, in the current study, we found that atomoxetine modestly, but significantly, increased impulsive choice. Apart from some methodological differences, only a single dose was tested in the present study and therefore future work employing multiple doses of atomoxetine should resolve this discrepancy. Collectively, the data obtained from the present rat studies strongly indicate that the currently employed measures of impulsive choice and impulsive action are separable both on a behavioral and neurobiological level, at least in terms of dopamine and norepinephrine neurotransmission.

In further support of the current pharmacological data, neuroanatomical evidence also reveals common as well as distinct neurocircuitries modulating impulsive choice and impulsive action in both rats (for reviews see [4], [8], [9]) and humans (for reviews see [10], [11]). Altogether, the data obtained from present and previous rat studies strongly indicate that the currently employed measures of impulsive choice and impulsive action are separable both on a behavioral and neurobiological level.

### Impulsive choice and action are unrelated in healthy volunteers

Consistent with the rodent data, our results in human volunteers yielded separate constructs of impulsive action, impulsive choice and self-reported impulsivity. These findings are consistent with previous human studies investigating which constructs of impulsivity can be dissected within subjects using correlation and principal component analyses on laboratory behavioral tasks [3], [21], [22], [24]. In these studies, impulsive choice was measured using delay discounting paradigms similar to the current study. However, impulsive action was examined with different measures across studies: whereas some studies only used the IMT/DMT or the CPT in order to measure impulsive action [22], others employed the SST or go-no go tasks only [3] or used both the IMT/DMT or the CPT and the SST or the go-no go task [21], [24]. Therefore, we included both the IMT/DMT and the SST to examine whether these tasks measure the same factor (impulsive action) or represent different behavioral outcomes as previously reported [21], [24]. In the present study, the IMT and DMT were found to load on a single component (impulsive action). The loading of the SST (0.48) almost met the criterion to be considered as a relevant loading on this component (>0.5) and we found a significant but modest correlation between the DMT and SST measures. Previous work [43] investigating multiple aspects of impulsivity in adolescents with disruptive behavior disorders yielded similar results in relation to the go-no go task, namely one component consisting of delayed reward measures and one component including the IMT and DMT with high loadings (>0.80) and a Go/Stop paradigm with a lower loading (around 0.50). It should be noted that although both the IMT/DMT and SST are considered measures of impulsive action, these tasks differ in at least one important aspect. In the IMT/DMT, subjects have to refrain from responding until the target stimulus is accurately processed in order to prevent impulsive, incorrect responses; a type of impulsivity also referred to as ‘action restraint’ [44]. In the SST, on the other hand, subjects already initiated their response and have to cancel this response whenever a stop signal is presented. This type of impulsivity is also called ‘action cancelation’ [44]. Unexpectedly, we found a negative loading of the SST on the impulsive choice (DDT) factor. Instead of no relation between SST and DDT which was expected, the SST and DDT were actually inversely related to each other. Clearly, the modest relation between IMT/DMT and SST, and the negative relation between SST and DDT suggests that action restraint and action cancellation are more similar to one another than to impulsive choice, but are not identical. Therefore, when selecting behavioral measures of impulsivity, it should be taken into account that tasks measuring part of the same construct may still have subtle differences and assess different aspects of impulsivity. The DDT was found to load on a separate component, which is consistent with other studies using within-subjects comparisons to dissect several constructs of impulsivity [3], [21], [22], [24]. Interestingly, the SST showed a negative loading on this component, indicating an inverse impact of the SST on the factor of impulsive choice.

Similar to previous reports [3], [22], [24], [30], the subscales of the BIS-11 were associated with a separate component, indicating that there are fundamental differences between self-report measures and behavioral assessments. For instance, self-report measures rely on accurate assessments of someone's own behavior and are therefore prone to response bias. On the other hand, self-report questionnaires incorporate social aspects of impulsivity. For this reason, generalizability of conclusions from behavioral findings to broader behavioral contexts may be limited.

### Translational implications

To date, only a few rodent studies have tested both impulsive action and choice in the same animals [18]–[20] and their results have been inconsistent. Human studies using a within-subjects design to assess the multidimensional construct of impulsivity have yielded a clearer indication of separate constructs of impulsive action and impulsive choice [3], [21]–[24]. Investigating whether similar constructs can be identified in rodents is important, because these model systems allow to further elucidate the neurobiological mechanisms underlying (maladaptive) impulsive behavior as displayed in humans. The current study revealed that impulsive choice and impulsive action appear not to be correlated in both rats and humans when using similar behavioral tasks in both species. The cognitive paradigms employed in the current study were selected to allow direct comparison of measures of impulsive behavior in rats and human healthy volunteers. Although the 5-CSRTT was originally developed as an analogue of the continuous performance task [45], the standard continuous performance task readily suffers from ceiling effects in healthy subjects [31]. Therefore, in the current study, the IMT/DMT was used, a more demanding paradigm similarly assessing impulsive action [31]. With regard to the DRT employed in rats, this task is comparable to the DDT used in humans and the discounting curve (including k-value and indifference point) can be estimated by the same equation [26] in both species [46]. Interestingly, employing these cross-species analogous measures of impulsivity yielded similar results in both our rats and human volunteers, namely a lack of correlation between impulsive choice and impulsive action. Similar results across species are in line with these observations. Chamberlain et al. [47] reviewed the translational value of neuropsychological tests of the CANTAB battery related to ADHD and reported similar pharmacological effects on both human cognitive tests and their animal counterparts. Thus, these findings and the current results provide further support for implementing rodent behavioral measures to unravel the underlying neurobiological mechanisms of impulsivity and other executive cognitive domains.

Although no data were collected on other aspects of impulsivity (e.g. reflection impulsivity [48], [49]), the current study shows that impulsive action and impulsive choice are dissociable behavioral phenomena of impulsivity. This is important to acknowledge when investigating the role of impulsivity in psychiatric disorders, which may vary across disorders. For instance, the severity of antisocial personality disorder was shown to be strongly associated with maladaptive levels of impulsive action, and not impulsive choice [50]. Conversely, in many psychiatric disorders such as substance dependence, bipolar disorder and ADHD, both impulsive choice and impulsive action coexist. Nonetheless, it is important to note that the involvement of various aspects of impulsivity may vary across different stages or clinical manifestations of a particular disorder. For example, initial sensitivity to nicotine reward and reinforcement is predicted by impulsive action whereas impulsive choice predicts persistence of nicotine seeking and enhanced vulnerability to relapse in both rats and humans [14], [51], [52]. Also, children diagnosed with ADHD show both increased impulsive choice and impulsive action, though these measures did not correlate within individuals and are associated with different characteristics of ADHD [12]. This latter finding indicates that impulsive choice and impulsive action are not only unrelated in healthy subjects, but also constitute separate constructs in a disease state that is characterized by an overall higher and maladaptive level of impulsivity.

Our results should be viewed in light of some methodological limitations. Although we aimed to match the behavioural paradigms in the rodent and human study, caution is required when attempting to translate the current findings in rodents to the human data. For example, in the human study, hypothetical rewards were presented during the delay discounting task, whereas the rats instantly faced the consequence of their choice in the form of food pellets. Although there is evidence suggesting that comparable results are obtained in humans when using real or hypothetical rewards (e.g. [53]–[55]), one cannot rule out the possibility of different motivational processes involved in the animal and human study. In addition, there are obvious differences between the human IMT/DMT task and the rodent 5-CSRTT. In the 5-CSRTT, no stimulus is presented when a premature response is made, whereas in the IMT/DMT, a premature response is made in reaction to a stimulus and these stimuli are designed to be ambiguous. Therefore the stimuli in the IMT/DMT exert a higher cognitive load for the human subjects compared to the rodents. Notwithstanding these limitations, we believe that the impact of these considerations on the main findings is limited. Although the behavioral paradigms were not perfectly matched, the animal and the human study, both using a within-subjects design, yielded the same result, namely, a lack of correlation between impulsive choice and impulsive action.

Clearly, the current study suggests that the development of more efficient treatment strategies will benefit from taking into account the multidimensional nature of impulsivity as demonstrated here in a cross-species within-subjects approach. Examining the relative contribution of separate aspects of impulsivity to different stages or clinical manifestations of psychiatric disorders and the neurobiology underlying these distinct aspects of impulsivity could in future lead to the development of more specific and tailored pharmacotherapies to treat maladaptive impulsivity.

## Supporting Information

Methods S1 **Detailed description of Delayed Reward Task (DRT) and Five Choice Serial Reaction Time Task (5-CSRTT).**
(DOC)

Table S1 **The dependent measures of the DRT in rats (n = 22)**. Indifference points were increased by AMP and decreased by ATO. Omissions remained unchanged.
(DOC)

Table S2 **The dependent measures of the 5-CSRTT in rats (n = 22).** Lengthening the inter trial interval (from 5 to 7 s) increased the number of premature responses and decreased accuracy. AMP increased the number of premature responses and decreased accuracy. ATO decreased the number of premature responses. The other dependent measures remained unchanged.
(DOC)

Table S3 **The correlations between the various impulsivity measures in humans (N = 100).** The subscales of the BIS-11 correlated significantly with each other. In addition, there was a significant correlation between the IMT Ratio and DMT Ratio and between the DMT Ratio and Stop SSRT.
(DOC)

Table S4 **The demographic characteristics and the impulsivity scores in humans (N = 100).** There were no differences between males and females on any of the measures, indicating that gender had no influence on the correlation and factor analyses.
(DOC)
