# Supplementary material for: The Relationship between Impulsive Choice and Impulsive Action: A Cross-Species Translational Study
Source: PLoS One. 2012 May 4;7(5):e36781. doi: 10.1371/journal.pone.0036781 (PMC3344935; doi:10.1371/journal.pone.0036781)
Supplement: Methods S1 — Detailed description of Delayed Reward Task (DRT) and Five Choice Serial Reaction Time Task (5-CSRTT). (DOC) [file pone.0036781.s001.doc]

# Supporting methods

## Detailed description of Delayed Reward Task (DRT)

Training of rats occurred in 16 identical rat two lever operant chambers (Med Associates, St. Albans, VT, USA). A white house light was located above the food receptacle. On both sides of the food receptacle was a retractable lever. Above the food receptacle and levers were stimulus lights.

*Habituation* Rats were habituated to the training environment for 15 minutes. The house light was illuminated, and the pellet dispenser contained four pellets. Next, there were two sessions of 100 pellet deliveries, randomly delivered with an average interval of 15 seconds. During these sessions, the house light was on.

*Training phase one* Training sessions of phase 1 and 2 ended after 75 rewards or 30 minutes, whichever came first. The house light was illuminated during the entire session. Both levers were extended and the stimulus lights above were illuminated during the entire session. Every lever press was rewarded with a food pellet.

*Training phase two* Every trial started by turning on the cue light above the food receptacle. Once a nose poke into the receptacle was made this light was switched of, left and right cue-lights were illuminated and levers extended. When a lever press was made cue lights were extinguished, a food pellet was delivered and a new trial commenced.

*Training phase three* Training continued by introducing the choice between 1 and 4 pellets. Sessions contained 5 blocks of 12 trials. The first two trials of each block were forced choice trials. A trial started by illumination of the cue light above the receptacle. After a nose-poke was made, left or right lever was extended and the corresponding cue light was illuminated. A lever press was rewarded with 1 pellet on one side and with 4 pellets on the other side. The position of the small and large reward was always the same for one animal, but counterbalanced between animals. The forced trials were followed by 10 choice trials. These trials started with the illumination of the middle light, after a nose-poke was made, left and right levers were extended and corresponding cue-lights were illuminated. In this way rats could choose to press for 1 pellet on one side, or 4 pellets on the other side. Once a lever press was made, levers were retracted, cue lights were switched off and the corresponding reward was delivered. If the rat did not respond within 10 seconds, the levers were retracted, cue lights were switched off and the inter trial interval commenced.

*Training phase four* In this phase the delay for the large reward was introduced. There were again 5 blocks of 12 trials, including 2 forced and 10 choice trials. The forced trials indicated the length of the delay for the large reward. All trials were the same as during training phase three except that the large reward was provided after a specific delay. The small rewards were always delivered immediately. For the first two sessions the delay of the large reward increased over blocks as follows: 0, 1, 2, 4, and 8 seconds. During the following three sessions the delay increased from 0, 2, 4, and 8, to 16 seconds. Another three sessions were subsequently provided incorporating delays of 0, 4, 8, 16 and 32 seconds. Inter trial intervals of the whole session were adapted to the length of the longest delay of that session. In this way it was always more advantageous to choose for the larger reward.

*Final procedure* The entire procedure was identical to the last phase of training except for the delays between the lever press and the delivery of the large reward. In the final procedure, the delay of the large reward increased over blocks as follows: 0, 5, 10, 20, and 40 seconds. Dependent variables of the DRT were the percentage of omissions in choice trials, the preference for the large rewards for each separate delay and the indifference point. The indifference point was based on the equation of Mazur (1987): Indifference point = Preference at delay 0 / (1 + k*delay). Rats that showed less than 50% preference for the large reward at delay 0, or that did not show a decrease in preference over increasing delays were excluded from the analyses.

## Detailed description of the Five Choice Serial Reaction Time Task (5-CSRTT)

Training of rats occurred in 32 identical rat five-hole nose poke operant chambers (Med Associates, St. Albans, VT, USA). A cue light was located in each of the holes. A white house light was located above the food receptacle on the wall opposing the five nose-poke holes.

*Habituation* Rats were habituated to the training environment for 15 minutes. The house light was illuminated, and the pellet dispenser contained four pellets. Next, there were two sessions of 100 pellet deliveries, randomly delivered with an average interval of 15 seconds. During these sessions, the house light was on.

*Training phase one* Sessions ended after 75 rewards or 30 minutes, whichever came first. The house light was illuminated during the entire session. All five cue lights in the nose-poke holes were illuminated. When a nose-poke into one of these 5 holes was made a food pellet was delivered and all lights were extinguished for 1 second.

*Training phase two* Sessions ended after 75 rewards or 30 minutes, whichever came first. The house light was illuminated during the entire session. Every trial was started by random illumination of one of the five cue lights. Once a nose poke into this hole was made a food pellet was delivered, the cue light was switched off and a new trial commenced.

*Training phase three* Sessions ended after 100 trials or 30 minutes, whichever came first. Every trial started by a nose-poke into the food receptacle, and an inter trial interval of 5 seconds. After these 5 seconds one of the five cue lights was illuminated. The cue presentation was at first set at 32 seconds and was gradually (16, 8, 4, 2 and 1.5 s) decreased over sessions to 1 s in the final stage of the 5-CSRTT. A correct response during stimulus presentation or within a 2 s limited hold period was rewarded with a food pellet and the stimulus light was extinguished. Omissions and premature responses (responses during the inter trial interval, before cue presentation) were punished with a time out. This 5 s time out was signaled by extinction of the house light.

*Final procedure* The final procedure of the 5-CSRTT was as described in training phase 3 with a cue presentation of 1 s. The standard inter trial interval (ITI) was 5 s. At the end of training, there were 3 sessions with a lengthened ITI of 7 s. There was one week with normal ITI training in between these sessions. Dependent variables are the number of premature responses (impulsive action), the percentage of correct responses (accuracy), the number of omissions, latency to make a correct response and the number of perseverative responses after a correct response. Rats showing an accuracy of less than 70%, or more than 20% omissions during baseline sessions were excluded from the analyses.
