# Supplementary material for: The Relationship between Impulsive Choice and Impulsive Action: A Cross-Species Translational Study
Source: PLoS One. 2012 May 4;7(5):e36781. doi: 10.1371/journal.pone.0036781 (PMC3344935; doi:10.1371/journal.pone.0036781)
Supplement: Table S1 — The dependent measures of the DRT in rats (n = 22). Indifference points were increased by AMP and decreased by ATO. Omissions remained unchanged. (DOC) [file pone.0036781.s002.doc]

*Table S1: Delayed Reward Task (N=22)*

|  | Indifference point | | Percentage omissions | |
| --- | --- | --- | --- | --- |
|  | Mean | SEM | Mean | SEM |
| Baseline | 21.5 | ± 4.2 | 3.6 | ± .84 |
| Saline | 18.6 | ± 3.7 | 2.1 | ± .78 |
| AMP | 29.6 °° | ± 4.0 | 2.2 | ± .70 |
| ATO | 12.8 ° | ± 3.2 | 2.7 | ± 1.1 |

Description of performance in the delayed reward task. Mean ± SEM; °p<0.05, °°p<0.001 vs saline.
