# Supplementary material for: The Relationship between Impulsive Choice and Impulsive Action: A Cross-Species Translational Study
Source: PLoS One. 2012 May 4;7(5):e36781. doi: 10.1371/journal.pone.0036781 (PMC3344935; doi:10.1371/journal.pone.0036781)
Supplement: Table S2 — The dependent measures of the 5-CSRTT in rats (n = 22). Lengthening the inter trial interval (from 5 to 7 s) increased the number of premature responses and decreased accuracy. AMP increased the number of premature responses and decreased accuracy. ATO decreased the number of premature responses. The other dependent measures remained unchanged. (DOC) [file pone.0036781.s003.doc]

*Table 2: Five choice serial reaction time task (N=22)*

|  | Premature | | Omissions | | Accuracy | | Latency correct | | Perseverative | |
| --- | --- | --- | --- | --- | --- | --- | --- | --- | --- | --- |
|  | Mean | SEM | Mean | SEM | Mean | SEM | Mean | SEM | Mean | SEM |
| ITI5 | 14.5 | ± 2.3 | 13.6 | ± 1.9 | 81.0 | ± 1.5 | .34 | ± .01 | 6.8 | ± .92 |
| ITI 7 | 57.4 ** | ± 4.1 | 12.4 | ± 1.6 | 74.8 ** | ± 1.4 | .32 | ± .01 | 5.6 | ± .81 |
|  |  |  |  |  |  |  |  |  |  |  |
| Saline | 19.8 | ± 3.2 | 11.0 | ± 1.6 | 81.0 | ± 1.8 | .35 | ± .01 | 10.7 | ± 2.0 |
| AMP | 79.4 °° | ± 8.7 | 10.1 | ± 1.9 | 74.5 ° | ± 2.1 | .33 | ± .02 | 10.2 | ± 2.0 |
| ATO | 10.6 ° | ± 1.6 | 16.0 | ± 3.6 | 84.1 | ± 1.9 | .38 | ± .02 | 10.2 | ± 2.2 |

Description of performance in the five-choice serial reaction time task. Mean ± SEM; * p<0.05, **p<0.001 vs ITI 5; °p<0.05, °°p<0.001 vs saline.
