# Supplementary material for: The Relationship between Impulsive Choice and Impulsive Action: A Cross-Species Translational Study
Source: PLoS One. 2012 May 4;7(5):e36781. doi: 10.1371/journal.pone.0036781 (PMC3344935; doi:10.1371/journal.pone.0036781)
Supplement: Table S3 — The correlations between the various impulsivity measures in humans (N = 100). The subscales of the BIS-11 correlated significantly with each other. In addition, there was a significant correlation between the IMT Ratio and DMT Ratio and between the DMT Ratio and Stop SSRT. (DOC) [file pone.0036781.s004.doc]

*Table S3: Correlations between impulsivity measures (N=100*)

| *Correlation matrix* |  | | | | | |
| --- | --- | --- | --- | --- | --- | --- |
|  | *IMT Ratio^b^* | *DMT Ratio^b^* | *Stop*  *SSRT* | *BIS-11*  *Cognitive* | *BIS-11 motor* | *BIS-11 non-planning* |
| DDT k valueª | 0.11 | 0.16 | 0.04 | -0.11 | -0.02 | 0.13 |
| IMT Ratio^b^ |  | 0.64** | 0.17 | -0.07 | 0.11 | 0.09 |
| DMT Ratio^b^ |  |  | 0.20* | -0.04 | -0.02 | 0.12 |
| Stop SSRT |  |  |  | 0.07 | 0.05 | -0.06 |
| BIS-11 cognitive impulsivity |  |  |  |  | 0.42** | 0.34** |
| BIS-11 motor impulsivity |  |  |  |  |  | 0.38** |

* Significance p<0.05

** Significance p<0.001

ª k values were obtained by a hyperbolic decay function and log transformed

^b^ IMT and DMT scores were calculated as the ratio of commission errors to correct detections

*DDT: Delay Discounting Task, IMT: Immediate Memory Task, DMT: Delayed Memory Task,*

*SSRT: Stop Signal Reaction Time, BIS-11: Barratt Impulsiveness Scale*
