# Supplementary material for: The Relationship between Impulsive Choice and Impulsive Action: A Cross-Species Translational Study
Source: PLoS One. 2012 May 4;7(5):e36781. doi: 10.1371/journal.pone.0036781 (PMC3344935; doi:10.1371/journal.pone.0036781)
Supplement: Table S4 — The demographic characteristics and the impulsivity scores in humans (N = 100). There were no differences between males and females on any of the measures, indicating that gender had no influence on the correlation and factor analyses. (DOC) [file pone.0036781.s005.doc]

*Table S4: Demographics and impulsivity scores (N=100*)

|  | Females (N=78) | | Males (N=22) | |
| --- | --- | --- | --- | --- |
|  | Mean | SEM | Mean | SEM |
| Age | 21.18 | ± 0.28 | 21.09 | ± 0.45 |
| Cigarettes/week | 3.04 | ± 1.56 | 2.18 | ± 1.92 |
| Alcohol in standard units/week | 4.39 | ± 0.49 | 6.52 | ± 1.49 |
| DDT k valueª | -5.34 | ± 0.20 | -6.07 | ± 0.36 |
| IMT Ratio ^b^ | 0.34 | ± 0.02 | 0.28 | ± 0.03 |
| DMT Ratio ^b^ | 0.29 | ± 0.02 | 0.26 | ± 0.03 |
| Stop SSRT | 285.00 | ± 5.12 | 274.25 | ± 6.00 |
| BIS-11 total score | 61.44 | ± 0.99 | 60.36 | ± 1.93 |
| BIS-11 cognitive impulsivity | 16.59 | ± 0.45 | 17.09 | ± 0.88 |
| BIS-11 motor impulsivity | 21.68 | ± 0.46 | 21.27 | ± 0.87 |
| BIS-11 non-planning impulsivity | 23.17 | ± 0.41 | 22.00 | ± 0.79 |

ª k values were obtained by a hyperbolic decay function and log transformed

^b^ IMT and DMT scores were calculated as the ratio of commission errors to correct detections

*DDT: Delay Discounting Task, IMT: Immediate Memory Task, DMT: Delayed Memory Task,*

*SSRT: Stop Signal Reaction Time, BIS-11: Barratt Impulsiveness Scale*
